# Knowledge, Attitude, and Early Detection Practices of Breast Cancer Among Employed Women in Selected Cities of the Amhara Regional State, Ethiopia: A Cross‐Sectional Study

**DOI:** 10.1002/hsr2.72836

**Published:** 2026-07-19

**Authors:** Abaineh Munshea, Bizuayehu Kerisew, Endalkachew Nibret, Mastewal Alehegn, Belaynesh Tazebew, Alemayehu Abate

**Affiliations:** ^1^ Health Biotechnology Division, Institute of Biotechnology Bahir Dar University Bahir Dar Ethiopia; ^2^ Biology Department Bahir Dar University Bahir Dar Ethiopia; ^3^ Research Development Directorate Amhara Public Health Institute Bahir Dar Ethiopia

**Keywords:** awareness, breast cancer, early detection, Ethiopian women, risk factors, screening

## Abstract

**Background and Aims:**

Breast cancer is the most common malignancy among women globally and a leading cause of cancer‐related mortality in Ethiopia. Early detection and awareness of risk factors are critical for improving outcomes, yet knowledge gaps and sociocultural barriers persist. The objective of this study was to assess awareness of breast cancer risk factors, symptoms, diagnostic methods, and preventive practices among Ethiopian women.

**Methods:**

A community‐based cross‐sectional study was conducted from September 2024 to May 2025 among women employed in governmental, non‐governmental, and private organizations in Bahir Dar, Dessie, Debre Berhan, and Gondar. A multistage random sampling technique selected 473 participants. Data were collected using a semi‐structured questionnaire covering socio‐demographic characteristics, knowledge of breast cancer, attitudes toward screening, and early detection practices. Data were analyzed using SPSS version 20.0 and Epi Info 3.5.1. Binary and multivariable logistic regression analyses were performed to identify factors associated with knowledge, attitude, and practice. Adjusted odds ratios (AORs) with 95% confidence intervals were used to determine the strength of associations, and statistical significance was declared at *p* < 0.05.

**Results:**

Participants were predominantly young (≤ 30 years) and well‐educated (48.0% diploma, 41.2% BSc or higher). Commonly recognized symptoms were breast pain (86.3%), swelling (83.3%), lumps (79.5%), and size changes (78.4%). Self‐breast examination was practiced by 68.7%, but knowledge of correct timing and frequency was low. Awareness of Clinical‐breast examination (88.6%) and pathological diagnosis (54.1%) was higher. Risk factor knowledge was uneven, with better recognition of smoking, alcohol, and inactivity, but poor understanding of diet, obesity, reproductive factors, hormones, and radiation. Sunlight was commonly misperceived as a risk. Main information sources were mass media, health workers, and teachers. Participants aged 36–40 years had significantly better knowledge (AOR = 2.21, 95% CI: 1.51–3.14, *p* = 0.002). Family history of breast cancer was associated with higher odds of good knowledge (AOR = 2.13, 95% CI: 1.24–3.61, *p* = 0.004), good attitude (AOR = 2.35, 95% CI: 1.26–4.46, *p* = 0.004), and good practices (AOR = 1.64, 95% CI: 0.96–2.31, *p* = 0.041). Having a current breast problem was the strongest predictor, associated with better knowledge (AOR = 2.24, 95% CI: 1.48–3.84, *p* = 0.004), better attitude (AOR = 3.64, 95% CI: 2.62–8.24, *p* = 0.001), and good preventive practices (AOR = 3.54, 95% CI: 1.87–6.25, *p* = 0.003).

**Conclusion:**

Breast cancer knowledge among Ethiopian women remains poor with notable gaps in understanding risk factors, symptoms, and preventive practices. Sociocultural and structural barriers further impede early detection. Implementing targeted, culturally sensitive health education, integrating breast cancer awareness into routine healthcare, and utilizing mass media and educational institutions are essential strategies to enhance knowledge, promote screening, and improve early detection, ultimately reducing morbidity and mortality.

## 1 Introduction

Breast cancer, a malignant disease of the breast's glandular tissues, is the most frequently diagnosed cancer in women worldwide, about 1 in 18 women develops it in her lifetime [1]. In 2022, it caused 2.3 million new cases and over 666,000 deaths, mostly in low‐ and middle‐income countries due to weak health systems [2]. Sub‐Saharan Africa alone reported about 146,130 new cases and 71,662 deaths in 2022 [2]. Low‐ and middle‐income countries, particularly in Africa, are seeing a rapid rise in breast cancer incidence due to population growth, aging, reproductive changes, and lifestyle factors such as obesity, inactivity, and hormonal contraception [3], 1.7 million new cases and 522,000 deaths were reported, with over half occurring in developing countries [4, 5, 6]. Low‐ and middle‐income countries, particularly in Africa, are experiencing a rapid rise in breast cancer incidence due to population growth, aging, reproductive changes, and lifestyle factors such as obesity, reduced physical activity, and oral contraceptive use [7, 8, 9].

Breast cancer risk is influenced by multiple factors. Gender remains the most significant, with women at far higher risk than men. Other risk factors include personal and family history, menstrual history, reproductive factors, and exposure to carcinogens [10]. Lifestyle factors such as nulliparity, late childbirth, limited breastfeeding, hormonal therapy, alcohol consumption, smoking, obesity, high‐fat diet, and reduced physical activity also contribute to elevated risk [11, 12].

Early detection remains the cornerstone of breast cancer control. Diagnosis at early stages significantly reduces mortality and improves survival, as demonstrated in developed countries, whereas delayed diagnosis in low‐resource settings leads to poorer outcomes due to limited screening, diagnostic, and treatment facilities [13]. Screening modalities include mammography, ultrasound, MRI, clinical breast examination (CBE), and self‐breast examination (SBE). While mammography is effective in well‐resourced settings, low‐cost methods like CBE and SBE are essential in resource‐limited regions [13].

SBE is a simple, safe, and cost‐free technique allowing women to monitor their breast health, enhancing early detection, especially in low‐resource settings [6, 14]. Its effectiveness depends on education, outreach, and regular practice [13, 15]. Despite modern screening advances, many early breast cancer cases are self‐detected, highlighting the continued importance of SBE [16, 17]. However, barriers such as fear, embarrassment, lack of time, insufficient privacy, and negative attitudes limit practice, and poor awareness often delay care‐seeking, reducing survival and treatment options [18, 19]. A 2024 study in Ethiopia found that only 40.1% of reproductive‐age women practiced self‐breast examination (SBE). Factors such as lower education levels, lower income, and lack of family history of breast cancer were associated with reduced SBE practices [20]. This study aimed to assess the knowledge, attitudes, and early screening practices of breast cancer among employed female workers in governmental, non‐governmental, and private sectors in selected cities of the Amhara region, Ethiopia.

## 2 Materials and Methods

### 2.1 Study Area, Design and Period

This study was conducted in the Amhara Regional State, one of the nine regional states of Ethiopia. The capital city, Bahir Dar, is located approximately 578 km northwest of Addis Ababa. Geographically, the region lies between 9°00′ to 13°45′ N latitude and 36°00′ to 40°30′ E longitude, situated on the northern plateau of Ethiopia. It is bordered by Tigray Region to the north, Afar Region to the east, Oromia Region to the south, and Benishangul‐Gumuz Region and Sudan to the west. A community‐based cross‐sectional study was conducted from September 2024 to May 2025.

### 2.2 Study Population

The source population consisted of all employed women working in governmental, non‐governmental, and private organizations located in Bahir Dar, Dessie, Debre Berhan, and Gondar. The study population included women from the source population who met the following inclusion criteria: Currently employed in a government office, non‐governmental organization, or private institution within the selected cities, aged 18 years or older and willing to provide informed consent.

### 2.3 Sample Size Determination

The sample size was calculated using a single population proportion formula [21] with the following assumptions: Confidence Interval (CI): 95%, Critical Value (*Z_a_*/_2_): 1.96, Margin of Error (*d*): 0.05, proportion (*p*): 0.5, due to lack of prior data from the area. A design effect of 1.11 was used to account for the potential clustering effect and increased variance associated with the sampling design. After applying a design effect of 1.11 to account for sampling variation, and adding 10% to compensate for possible non‐response or incomplete data, the final sample size was 473. The design effect (DEFF) is commonly calculated using the formula: DEFF = 1 + (m – 1) ρ, where *m* represents the average cluster size and *ρ* (rho) represents the intraclass correlation coefficient (ICC) [22].

### 2.4 Sampling Procedure

A multistage sampling technique was used. In the first stage, organizations in the Amhara Regional State were stratified into governmental, non‐governmental, and private sectors and selected by simple random sampling. In the second stage, the number of participants per organization was proportionally allocated based on the total number of eligible female employees. In the final stage, individual female workers were selected using simple random sampling from the prepared sampling frame until the required sample size was achieved. A design effect was applied to the sample size calculation to account for clustering.

### 2.5 Data Collection Instrument and Procedure

Data were collected using a semi‐structured questionnaire comprising both closed‐ and open‐ended questions, designed to address the objectives of the study. The questionnaire was adapted from previous studies conducted on a similar topic (2; 4;7; 12, 21) and customized accordingly. Data collection was conducted by trained interviewers to ensure accuracy and consistency. Interviewers provided guidance as needed to clarify questions and facilitate complete responses, minimizing potential bias and maximizing data reliability.

### 2.6 Knowledge, Attitude, and Practice (KAP) Scoring System

Knowledge, attitude, and practice were assessed using structured questions with dichotomous response options (Yes/No). For each item, a correct response was assigned a score of Yes, while an incorrect or undesirable response was assigned a score of No. KAP categories were defined based on participants' responses to domain‐specific items. The scores for all items within each domain were summed to generate a total score for each participant. Following score computation, the total score of the study population was calculated separately for knowledge, attitude, and practice domains.

### 2.7 Data Analysis and Interpretation

Data was coded and entered into Epi Info 3.5.1 and analyzed using Statistical Package for the Social Sciences (SPSS) version 20.0. Descriptive statistics was used to summarize socio‐demographic characteristics and study variables. Binary and multivariable logistic regression analyses were performed to identify factors associated with knowledge, attitude, and practice. Adjusted odds ratios (AORs) with 95% confidence intervals (CIs) were used to determine the strength of associations, and statistical significance was declared at *p* < 0.05. Multicollinearity among independent variables was assessed using variance inflation factors (VIFs), with values < 10 indicating no significant multicollinearity. The Hosmer–Lemeshow goodness‐of‐fit test was conducted, with a non‐significant *p*‐value (*p* > 0.05).

### 2.8 Reliability and Validity of the Study

To ensure reliability and validity of information: Before the data collection period, training was given to data collectors, the questionnaires was checked to ensure quality, clarity, and completeness, and pretested for validity and reliability. The data collection was supervised by principal investigator, and 10% of the completed questionnaires was randomly selected and rechecked blindly to ensure quality control.

### 2.9 Ethical Considerations

The study protocol together with consent form was submitted to ethical review board of Bahir Dar University for possible approval. Ethical clearance with ethical approval number 42/2024 was obtained, letter of support was written to concerned offices in the selected cities of the region. Before collecting the relevant data, the subjects were informed about the study and written informed consents were obtained from all of the participants. Participation in the study was on voluntary basis and the study subjects were free to withdraw from the study before and after interview.

## 3 Results

### 3.1 Socio‐Demographic Characteristics of Respondents

A total of 473 participants were included in the study, with ages ranging from 19 to 65 years (mean = 29.9, SD = 7.2. The majority aged between 26 and 30 years (36.4%), followed by those aged ≤ 25 years (30.0%). Participants aged 31–35, 36–40, 41–45, and > 45 years accounted for 16.9%, 9.9%, 3.0%, and 3.8%, respectively (Table 1).

**Table 1:** Socio‐demographic characteristics of respondents.

Variable	Category	Frequency	Percentage (%)
Age (years)	≤ 25	142	30.0
	26–30	172	36.4
	31–35	80	16.9
	36–40	47	9.9
	41–45	14	3.0
	> 45	18	3.8
Educational status	Grade 10 completed	18	3.8
	Grade 12 completed	18	3.8
	Certificate	15	3.2
	Diploma	227	48.0
	BSc and above	195	41.2
Marital status	Single	143	30.2
	Married	289	61.1
	Separated/Divorced	22	4.7
	Living with partner	13	2.7
	Widow	6	1.3
Family history of breast cancer	Yes	45	9.5
	No	428	90.5
Current breast problem	Yes	41	8.7
	No	432	91.3

### 3.2 Knowledge of Breast Cancer Risk Factors Among Participants

The study assessed participants’ awareness of various breast cancer risk factors. A majority (70.0%) recognized exposure to sunlight (a misconception) as a potential risk factor. Knowledge as a risk factor was higher for modifiable lifestyle factors such as cigarette smoking (63.0%), alcohol consumption (53.7%), and inactivity or sedentary lifestyle (50.1%). Over all the knowledge of participants on breast cancer potential risk factors was poor, 81%; 384/473 (Table 2).

**Table 2:** Knowledge on potential risk factors related to breast cancer among respondents.

Variable	Category	Frequency	Percentage (%)
Exposure to sun	Yes	331	70.0
	No	142	30.0
High fat diet	Yes	171	36.2
	No	302	63.8
Exposure to X‐ray before 30 years	Yes	226	47.8
	No	247	52.2
Overweight after menopause	Yes	139	29.4
	No	334	70.6
Early use of oral contraceptives	Yes	167	35.3
	No	306	64.7
Stress	Yes	204	43.1
	No	269	56.9
Alcohol consumption	Yes	254	53.7
	No	219	46.3
Cigarette smoking	Yes	298	63.0
	No	175	37.0
Inactivity and sedentary lifestyle	Yes	237	50.1
	No	236	49.9
Older age	Yes	219	46.3
	No	254	53.7
Family history of breast cancer	Yes	233	49.3
	No	240	50.7
Having first child at late age	Yes	153	32.3
	No	320	67.7
Early onset of menarche	Yes	103	21.8
	No	370	78.2
Late menopause	Yes	115	24.3
	No	358	75.7
Race/Ethnicity	Yes	166	35.1
	No	307	64.9

### 3.3 Knowledge on Breast Cancer Signs and Symptoms Among Respondents

The majority of participants reported being aware of key breast cancer symptoms. Knowledge of pain or soreness in the breast was the most common, reported by 86.3% of respondents. Followed by knowledge of swelling or enlargement of the breast (83.3%), a lump in the breast (79.5%), and changes in breast size (78.4%) (Table 3).

**Table 3:** Awareness on signs and symptoms of breast cancer among Respondents.

Variable	Category	Frequency	Percentage (%)
Lump in the breast	Yes	376	79.5
	No	97	20.5
Weight loss	Yes	253	53.5
	No	220	46.5
Discharge from the breast	Yes	333	70.4
	No	140	29.6
Pain or soreness in the breast	Yes	408	86.3
	No	65	13.7
Change in the size of the breast	Yes	371	78.4
	No	102	21.6
Discoloration/dimpling of the breast	Yes	314	66.4
	No	159	33.6
Ulceration of the breast	Yes	353	74.6
	No	120	25.4
Change in the shape of the breast	Yes	354	74.8
	No	119	25.2
Swelling/enlargement of the breast	Yes	394	83.3
	No	79	16.7
Lump under the armpit	Yes	267	56.4
	No	206	43.6
Scaling/dry skin in the nipple region	Yes	249	52.6
	No	224	47.4

### 3.4 Knowledge on Breast Cancer Screening and Diagnostic Practices Among Respondents

Knowledge of diagnostic methods among the participants, 58.1% reported performing self‐breast examination (SBE) as a diagnosis of breast cancer, while 41.9% did not. Clinical breast examination (CBE) performed by a physician was reported as the primary method of breast cancer diagnosis, with 88.6% of respondents indicating its use. Pathological examination as a diagnosis of breast cancer was reported by 54.1% of participants (Table 4).

**Table 4:** Knowledge on breast cancer screening and diagnostic practices among respondents.

Variable	Category	Frequency	Percentage (%)
Pathological Examination (*screening)*	Yes	256	54.1
	No	217	45.9
Self‐Breast Examination (Diagnostic)	Yes	275	58.1
	No	198	41.9
Clinical Breast Examination by Doctor (*screening)*	Yes	419	88.6
	No	54	11.4
Mammography (Diagnostic)	Yes	198	41.9
	No	275	58.1
Ultrasound (Diagnostic)	Yes	346	73.2
	No	127	26.8
Genetic Analysis (Diagnostic)	Yes	176	37.2
	No	297	62.8

### 3.5 Knowledge and Awareness of Self‐Breast Examination (SBE) Among Respondents

Among the study participants, 64.9% had heard about SBE, while 35.1% had not. Regarding understanding of SBE, 36.6% correctly identified it as a technique to help women know the shape and size of their breasts, 27.1% recognized it as a method for detecting breast lumps, 8.9% believed it was an assessment done by doctors or nurses, 1.7% thought it was for cosmetic purposes, and 25.8% selected all options (Table 5).

**Table 5:** Knowledge and awareness of self‐breast examination (SBE) among Respondents.

Variable	Category	Frequency	Percentage (%)
Heard about SBE	Yes	307	64.9
	No	166	35.1
Understanding of SBE	Technique for detecting breast lump	128	27.1
	Assessment done by doctors/nurses	42	8.9
	Technique helping women know breast shape/size	173	36.6
	Cosmetic purpose	8	1.7
	All of the above	122	25.8
Knowledge on how to perform SBE	Yes	221	46.7
	No	252	53.3
Knowledge on age to Start SBE	Yes	171	36.2
	No	302	63.8
Knowledge on frequency of SBE	Daily	73	15.4
	Weekly	71	15.0
	Monthly	55	11.6
	Undefined	48	10.1
	Don't know	226	47.8

### 3.6 Practice, Reasons, and Barriers Related to SBE Among Respondents

In this study, 68.7% (*n* = 211) reported practicing SBE, while 31.3% (*n* = 96) did not. Of those who practiced SBE, 53.7% performed it once a month, 15.6% once every 3 months, 11.1% more than once in 3 months, 13.7% not very often, and 5.9% greater than once a year.

SBE was most commonly initiated before the age of 25 years (40.4%), followed by ages 25–30 (29.0%), over 35 years (22.8%), and 30–35 years (7.8%) (Table 6).

**Table 6:** Practice, reasons, and barriers related to self‐breast examination (SBE).

Variable	Category	Frequency	Percentage (%)
Practice of SBE	Yes	211	68.7
	No	96	31.3
Frequency of SBE practice	Once a month	165	53.7
	Once in 3 months	48	15.6
	More than once in 3 months	34	11.1
	Not very often	42	13.7
	Greater than once a year	18	5.9
Age at starting SBE	< 25 years	124	40.4
	25–30 years	89	29.0
	30–35 years	24	7.8
	> 35 years	70	22.8
Reasons for not practicing SBE regularly	No breast problem	157	51.1
	Do not think I should	48	15.6
	Not comfortable doing it	15	4.9
	Do not know how to perform	49	16.0
	Carelessness	18	5.9
	Too frequent to practice	8	2.6
	Unsure about its benefit	7	2.3
Reasons for practicing SBE	Fear of breast cancer/other diseases	148	48.2
	Family history of breast cancer	38	12.4
	Unusual breast appearance	98	31.9
	Others	23	7.5
Barriers to performing SBE	Lack of privacy	27	8.8
	Pressure of work	23	7.5
	Doubt about effectiveness	58	18.9
	Absence of symptoms/disease	158	51.5
	Forgetfulness	41	13.4

### 3.7 Attitude Towards Breast Cancer Among Respondents

Among the respondents, 66.4% (*n* = 204) believed that breast cancer is a curable disease, while 33.6% (*n* = 103) did not. A majority (67.1%, *n* = 206) reported that they would feel scared if they developed breast cancer, whereas 24.4% would not and 11.6% were unsure. Regarding health‐seeking behavior, 90.2% (*n* = 277) indicated that they would consult a doctor if they developed breast cancer, 7.2% would not, and 2.6% were unsure (Table 7).

**Table 7:** Perceptions and attitudes toward breast cancer among respondents.

Variable	Category	Frequency	Percentage (%)
Do you think breast cancer is a curable disease?	Yes	204	66.4
	No	103	33.6
If you develop breast cancer, will you be scared?	Yes	206	67.1
	No	75	24.4
	Don't know	55	11.6
If you develop breast cancer, will you consult a doctor?	Yes	277	90.2
	No	22	7.2
	Don't know	8	2.6
If you develop breast cancer, will you use traditional medicine?	Yes	45	14.7
	No	230	74.9
	Don't know	32	10.4
If you develop breast cancer, will you go to a prayer house?	Yes	253	82.4
	No	42	13.7
	Don't know	12	3.9
If you develop breast cancer, will you agree to perform mastectomy?	Yes	174	56.7
	No	84	27.4
	Don't know	49	16.0
Early detection of breast cancer improves chances of survival	Yes	237	77.2
	No	39	12.7
	Don't know	31	10.1
If you develop a breast lump, how soon will you see a doctor?	Within one week	237	77.2
	Within one month	34	11.1
	Within 1–3 months	18	5.9
	Not bother at all	18	5.9
Will you allow a male doctor to examine your breast?	Yes	259	84.4
	No	48	15.6
Do you think long‐term survival (> 5 years) is rare due to breast cancer?	Yes	135	44.0
	No	172	56.0

### 3.8 Clinical Breast Examination (CBE) Practices and Reasons for Participation or Non‐Participation

Among the participants, only 35.2% (*n* = 108) reported ever having their breasts examined by a doctor, whereas 64.8% (*n* = 199) had never undergone a clinical breast examination. Among those who had been examined, 47.6% had a single examination, 27.4% had 1–3 examinations, 16.0% had 3–5 examinations, and 9.4% had more than five examinations (Table 8).

**Table 8:** Clinical breast examination (CBE) practices and reasons for participation or non‐participation.

Variable	Category	Frequency	Percentage (%)
Have you ever had your breast examined by a doctor?	Yes	108	35.2
	No	199	64.8
If Yes, Frequency of Examination	Once	146	47.6
	1–3 times	84	27.4
	3–5 times	49	16.0
	> 5 times	29	9.4
If No, Reasons for Reluctance to Perform CBE	Concern about extra cost	12	3.9
	Concern about extra time	8	2.6
	Fear of outcome	9	2.9
	Too young to participate	8	2.6
	No sign/symptom of breast cancer	223	72.6
	No one recommended it	18	5.9
	Unsure about benefit	23	7.5
	Other (specified)	6	2.0

### 3.9 Sources of Information About Breast Cancer Among Respondents

The majority of participants (67.8%, *n* = 208) reported obtaining information about breast cancer from television and radio. Other commonly cited sources included health workers (38.4%, *n* = 118) and school teachers (36.2%, *n* = 111) (Table 9).

**Table 9:** Sources of information about breast cancer among respondents.

Variable	Category	Frequency	Percentage (%)
Television and radio	Yes	208	67.8
	No	99	32.2
Friends	Yes	68	22.1
	No	239	77.9
Family	Yes	53	17.3
	No	254	82.7
Health workers	Yes	118	38.4
	No	189	61.6
School teachers	Yes	111	36.2
	No	196	63.8
Magazines and newspapers	Yes	25	8.1
	No	282	91.9
Internet	Yes	66	21.5
	No	241	78.5

### 3.10 Lifestyle and Preventive Practices Related to Breast Cancer Among Respondents

Most participants engaged in preventive behaviors, including regular self‐examination (78.8%, *n* = 242), and an increase in the frequency of self‐examination (75.9%, *n* = 233). Regarding lifestyle modifications, 61.6% (*n* = 189) had quit smoking, 77.5% (*n* = 238) avoided or limited alcohol consumption, 70.0% (*n* = 215) reduced dietary fat intake, and 80.8% (*n* = 248) increased their intake of fruits and vegetables (Table 10).

**Table 10:** Practices and lifestyle factors related to breast cancer prevention.

Variable	Category	Frequency	Percentage (%)
Frequent X‐rays (various body parts)	Yes	119	38.8
	No	188	61.2
Increase in Self‐Examination	Yes	233	75.9
	No	74	24.1
Smoking Cessation	Yes	189	61.6
	No	118	38.4
Avoidance/Limitation of Alcohol	Yes	238	77.5
	No	69	22.5
Decrease in Fat Intake	Yes	215	70.0
	No	92	30.0
Increase in Fruit and Vegetable Intake	Yes	248	80.8
	No	59	19.2
Use of Hormone Replacement Therapy	Yes	130	42.3
	No	177	57.7
Use of Hormonal Contraception	Yes	108	35.2
	No	199	64.8

### 3.11 Factors Associated With Knowledge, Practice, and Attitude Towards Breast Cancer

In multivariate analysis, participants aged 36–40 years were 2.21 times more likely to have good knowledge of breast cancer compared with those aged ≤ 25 years (AOR = 2.21, 95% CI: 1.51–3.14, *p* = 0.002). Respondents with a family history of breast cancer had higher odds of good practice toward breast cancer detection and prevention compared with those without a family history (AOR = 1.64, 95% CI: 0.96–2.31, *p* = 0.041 Participants with a family history of breast cancer were more likely to have good attitude toward breast cancer than those without a family history (AOR = 2.35, 95% CI: 1.26–4.46, *p* = 0.004). The details are presented in Table 11.

**Table 11:** Factors associated with knowledge, practice, and attitude towards breast cancer.

Outcome variable	Predictor variable	Category	Good	Poor	COR (95% CI)	*p*‐value	AOR (95% CI)	*p*‐value
Knowledge on Breast Cancer	Age (years)	≤ 25	42	100	Ref			
		26–30	48	124	0.92 (0.56–1.51)	0.743	—	—
		31–35	31	49	1.51 (0.85–2.68)	0.163	—	—
		36–40	28	19	3.51 (1.77–6.96)	< 0.001	2.21 (1.51–3.14)	0.002
		41–45	8	6	3.17 (1.04–9.72)	0.042	2.06 (1.46–5.27)	0.74
		> 45	11	7	3.74 (1.36–10.32)	0.011	2.31 (1.62–5.42)	0.53
	Educational Status	Grade 10 completed	6	12	Ref			
		Grade 12 completed	8	10	1.60 (0.41–6.17)	0.497	—	—
		Certificate	6	9	1.33 (0.32–5.53)	0.693	—	—
		Diploma	82	145	1.13 (0.41–3.13)	0.812	—	—
		BSc and above	66	129	1.02 (0.37–2.85)	0.965	—	—
	Marital Status	Single	55	88	Ref			
		Married	98	191	0.82 (0.54–1.24)	0.35	—	—
		Separated/Divorced	7	15	0.75 (0.29–1.95)	0.549	—	—
		Living with partner	6	7	1.37 (0.44–4.29)	0.586	—	—
		Widow	2	4	0.80 (0.14–4.52)	0.8	—	—
	Family History of Breast Cancer	Yes	26	19	2.76 (1.47–5.19)	0.002	2.13 (1.24–3.61)	0.004
		No	142	286	Ref			
	Current Breast Problem	Yes	24	17	2.82 (1.45–5.48)	0.002	2.24 (1.48–3.84)	0.004
		No	144	288	Ref			
Practice Toward Detection and Prevention	Age (years)	≤ 25	22	20	Ref			
		26–30	30	18	1.23 (0.56–2.71)	0.604	—	—
		31–35	16	15	0.82 (0.34–1.97)	0.651	—	—
		36–40	18	10	1.64 (0.64–4.21)	0.303	—	—
		41–45	4	4	1.45 (0.34–6.19)	0.612	—	—
		> 45	6	5	1.11 (0.33–3.76)	0.866	—	—
	Educational Status	Grade 10 completed	3	3	Ref			
		Grade 12 completed	5	3	1.00 (0.16–6.25)	1	—	—
		Certificate	3	3	1.00 (0.16–6.25)	1	—	—
		Diploma	43	39	1.09 (0.47–2.51)	0.845	—	—
		BSc and above	42	24	2.33 (1.00–5.41)	0.049	—	—
	Marital Status	Single	26	29	Ref			
		Married	63	35	1.62 (0.89–2.95)	0.114	—	—
		Separated/Divorced	3	4	0.86 (0.17–4.36)	0.853	—	—
		Living with partner	4	2	1.77 (0.29–10.73)	0.533	—	—
		Widow	0	2	0.00 (Undefined)	0.999	—	—
	Family History of Breast Cancer	Yes	20	6	2.89 (1.15–7.27)	0.024	1.64 (0.96–2.31)	0.041
		No	76	66	Ref			
	Current Breast Problem	Yes	20	4	4.47 (1.47–13.60)	0.008	3.54 (1.87–6.25)	0.003
		No	76	68	Ref			
Attitude Toward Breast Cancer	Age (years)	≤ 25	21	21	Ref			
		26–30	30	18	1.27 (0.58–2.76)	0.551	—	—
		31–35	16	15	0.83 (0.34–2.00)	0.667	—	—
		36–40	18	10	1.71 (0.67–4.37)	0.264	—	—
		41–45	4	4	1.43 (0.34–6.07)	0.625	—	—
		> 45	6	5	1.12 (0.33–3.79)	0.855	—	—
	Educational Status	Grade 10 completed	3	3	Ref			
		Grade 12 completed	5	3	1.67 (0.36–7.80)	0.509	—	—
		Certificate	3	3	1.00 (0.18–5.56)	1	—	—
		Diploma	43	39	1.09 (0.46–2.59)	0.839	—	—
		BSc and above	41	25	1.71 (0.71–4.13)	0.231	—	—
	Marital Status	Single	25	30	Ref			
		Married	63	35	1.83 (1.01–3.31)	0.046	—	—
		Separated/Divorced	3	4	0.86 (0.17–4.36)	0.853	—	—
		Living with partner	4	2	2.33 (0.37–14.48)	0.367	—	—
		Widow	0	2	0.00 (Undefined)	> 0.99	—	—
	Family History of Breast Cancer	Yes	20	6	2.98 (1.18–7.50)	0.021	2.35 (1.26–4.46)	0.004
		No	75	67	Ref			
	Current Breast Problem	Yes	20	4	4.60 (1.52–13.94)	0.007	3.64 (2.62–8.24)	< 0.001
		No	75	69	Ref			

## 4 Discussion

The study included 473 participants, most of whom were young, with nearly two‐thirds aged ≤ 30 years. Almost half held a diploma (48.0%), while 41.2% had a BSc degree or higher, indicating a relatively well‐educated sample. Similar findings have been reported in Ethiopia, where higher educational attainment is associated with improved breast cancer awareness and preventive practices [23, 24]. A small proportion reported a family history of breast cancer (9.5%) or current breast problems (8.7%) similar to previous Ethiopian studies [25, 26]. Women with a family history or with breast symptoms at present are usually more likely to participate in screening and early detection activities [27].

In this study, the majority (70.0%) incorrectly identified sunlight exposure as a risk factor, despite evidence suggesting potential protective effects through vitamin D, though findings remain inconclusive [28, 29, 30]. This finding reflects a persistent misconception and suggests gaps in accurate health education, as sunlight is not established as a direct risk factor for breast cancer. Such misunderstandings may indicate the influence of informal information sources or limited access to evidence‐based health education. Only 36.2% recognized a high‐fat diet as a risk factor, consistent with low awareness reported across Africa and elsewhere [31, 32]. Awareness of overweight/obesity after menopause was also limited (29.4%), comparable to Lebanon (31.5%) and Poland (22%) [33, 34]. Knowledge of modifiable risks was higher for smoking (63.0%), alcohol (53.7%), and physical inactivity (50.1%). These patterns reflect global variation, with high awareness in Europe but lower recognition in the Middle East [31, 35, 36].

These findings suggest that participants had limited understanding of hormonal and metabolic pathways that contribute to breast cancer development, which are critical components of prevention messaging. In this study, awareness of family history and increasing age as risk factors was moderate, but very few participants recognized reproductive factors such as early menarche or late first childbirth, consistent with findings from China and Africa [37, 38]. Less than half of participants identified early exposure to X‐rays, and stress as risk factors. While awareness of ionizing radiation was low elsewhere [34], belief in stress as a major cause was high in Germany and across Europe, despite lack of scientific consensus [35, 39]. This suggests limited understanding of reproductive, environmental, and genetic determinants of breast cancer, which are often less emphasized in community health education compared to lifestyle risks. Overall, this study demonstrated that 81% (384/473) of participants had poor knowledge of risk factors that increase the risk of breast cancer. This finding was similar with a study conducted in Wadila District, Northeast Ethiopia that reported among women of reproductive age reported that 65.3% had poor knowledge of breast cancer overall, while 86.6% had poor knowledge specifically regarding breast cancer risk factors [20]. This poor level of awareness suggests inadequate health education and screening advocacy in the study setting.

In this study, most participants were aware of common breast cancer symptoms. Breast pain or soreness was most frequently identified, followed by swelling, a lump, and changes in size. These findings align with global evidence showing lumps as the most widely recognized symptom, often > 90% in high‐income countries [27, 40], while awareness in LMICs ranges from 70% to 85% [32]. Recognition of breast pain varied internationally, with 35% in India [41] and 68% in Saudi Arabia. Awareness of changes in breast size/shape (78.4%) was consistent with UK studies [40]. Participants also recognized signs such as changes in breast shape, ulceration, and dimpling, while fewer identified lumps under the armpit or nipple skin changes. Nipple discharge was reported by 70.4%, higher than in India and the Middle East (40%–60%) [33, 41]. Awareness of unexplained weight loss (53.5%) matched global estimates of 45%–60% [42]. These findings indicated that health education and cancer awareness campaigns often emphasize more advanced or less commonly discussed symptoms, leading to moderate awareness.

Knowledge of diagnostic methods showed 58.1% reporting self‐breast examination (SBE), 88.6% clinical breast examination (CBE), and 54.1% pathological examination. These rates are consistent with African and European studies [32, 35]. International comparisons show SBE awareness varies widely—from 51.6% in India to 79% in Saudi Arabia [43, 44]. CBE awareness was higher than in Ethiopia (62%) and Cameroon (43.1%) [26]. Knowledge of imaging‐based screening was moderate (73.2% ultrasound, 41.9% mammography), consistent with China and Ethiopia [1], while genetic testing was least known, reflecting findings from Kenya and global reviews [45, 46]. This finding indicated that many participants may have misconstrued self‐screening practices as formal diagnostic methods, moderate awareness of histopathological confirmation as the gold standard for diagnosis, relatively low awareness of mammography in detecting asymptomatic and early‐stage disease, and a significant gap in awareness of advanced diagnostic and risk stratification tools.

In this study, 64.9% of participants had heard of self‐breast examination (SBE), while 35.1% had not. Similar rates were reported in Ethiopia (73.7% awareness, 35.6% correct practice) [47] and India (62.5% awareness, 12.9% regular practice) [43]. In Nigeria, women often associated SBE with general health monitoring rather than cancer detection [23]. Understanding was limited: 36.6% identified SBE as monitoring breast shape/size, 27.1% as detecting lumps, and 8.9% mistakenly thought it was a clinical procedure. Comparable findings were seen in Morocco [48] and across Africa, where correct knowledge of monthly timing was often < 20% [26]. Only 36.2% of participants knew the correct age to begin SBE. Knowledge of frequency was poor: 15.4% indicated daily, 15.0% weekly, and 11.6% monthly. Ethiopian studies similarly reported confusion, with only 8.2% identifying age 20 as the correct starting point [4, 32], while 45% practiced it monthly [49]. This finding indicated that even among those who have heard of SBE, a significant proportion lack the necessary skills and procedural knowledge required for proper implementation, confusion about standard guidelines, and may lead either to over‐practice, anxiety, or complete non‐adoption of the behavior.

In this study, 68.7% reported practicing SBE. Of these, 53.7% did so monthly, while others practiced less consistently. These rates fall within the 30%–60% range common in African and international studies [26], with similar figures in Ethiopia (36%) [50], Saudi Arabia (38.5%) [44], and Turkey (41.6%) [51]. About 20.5% began before age 25, comparable to Ethiopia (median 27 years) [52] and Nigeria (mean 22.6 years) [23].

In this study, the key reasons for non‐practice included absence of breast problems (51.1%) and lack of knowledge (16%), while motivations included fear of cancer (48.2%), unusual breast appearance (31.9%), and family history (12.4%). These patterns mirror findings from Addis Ababa and Wollo University [4, 25]. Reported barriers included absence of symptoms (51.5%), doubt about effectiveness (18.9%), and lack of privacy (8.8%), consistent with Ethiopian studies [53]. These findings suggested that while SBE practice is relatively common, it is frequently irregular, symptom‐driven, and influenced by misconceptions about its preventive role. This indicates a gap between knowledge and appropriate health behavior, emphasizing the need for targeted education that not only promotes SBE but also clarifies its purpose as a routine, systematic, and preventive practice regardless of symptom presence.

Most respondents (66.4%) believed breast cancer is curable. A majority (67.1%) reported that they would feel scared if they developed breast cancer, whereas 24.4% would not and 11.6% were unsure, echoing fatalistic views reported in Nigeria [54]. While 90.2% would consult a doctor if they developed breast cancer, 14.7% would consider traditional medicine and 82.4% would seek support from a prayer house, reflecting patterns of dual reliance on formal and spiritual care in Ghana [55]. The findings of this study indicated that while attitudes toward biomedical care and early consultation are generally favorable, important challenges remain, particularly regarding fear, cultural and spiritual coping preferences, uncertainty about treatment options, and delayed care‐seeking among a minority of participants.

In this study, 56.7% of respondents indicated willingness to undergo mastectomy, comparable to 57% in Ethiopia [56], though reluctance is often linked to concerns about life impact [57]. Most participants would see a doctor within 1 week of detecting a breast lump, contrasting with systematic reviews in LMICs where delays commonly range 1–3 months [27]. Regarding prognosis, 56% believed long‐term survival (> 5 years) is possible, while 44% considered it rare, echoing limited awareness in Ethiopian studies [25].

In this study, only a minority had undergone clinical breast examination (CBE), with 64.8% reporting never having one. This is consistent with low uptake in Ethiopia (6.1%–21%), Egypt (22.4%), and across Africa (8.9%–48.9%) [26, 54, 58, 59]. Barriers included absence of symptoms (72.6%), uncertainty about benefits (7.5%), lack of recommendation (5.9%), cost (3.9%), fear of the outcome (2.9%), time constraints (2.6%), and feeling too young (2.6%). Similar obstacles, low risk perception, fear, and limited knowledge have been reported in Ethiopia and elsewhere [4, 25, 46, 56]. The results of this study revealed that although participants generally express willingness to seek medical care, actual engagement with clinical screening services remains low, largely due to misconceptions about disease an absence equating to absence of need for screening.

In this study, mass‐media were the leading sources of information, followed by health workers and teachers, with fewer citing family, friends, or the internet. Newspapers and magazines were reported least. This aligns with surveys in Northern Ethiopia, where media accounted for nearly 60% of information sources [60], and in Nigeria, where 67.5% relied on mass‐media [17]. Similar patterns have been observed in rural India and Kenya, where radio is particularly influential [46]. The findings demonstrated a strong dependence on mass media, particularly television and radio, for breast cancer information, with relatively limited contribution from healthcare providers, family networks, and digital platforms.

Preventive practices included regular self‐breast examination (78.8%), increased frequency of SBE (75.9%), alcohol avoidance (77.5%), fruit and vegetable intake (80.8%), reduced dietary fat (70.0%), and smoking cessation (61.6%). Comparable levels of awareness have been reported in Ethiopia [49, 53] and Jordan, where smoking was less often recognized as a risk factor (47.8%) [61]. Knowledge of specific risk factors was limited: 38.8% recognized frequent X‐ray exposure, 42.3% hormone replacement therapy, and 35.2% hormonal contraception. These figures reflect international evidence, including U.S. data, showing awareness of such associations remains below 50%. The findings suggested that while participants demonstrate relatively strong engagement in preventive lifestyle behaviors and self‐screening practices, there are important gaps in understanding and interpretation of medical and exposure‐related risk factors.

The findings of this study showed a clear knowledge to practice discrepancy, where participants demonstrated acceptable or even good knowledge but did not consistently translate it into correct or sustained practice. The discrepancy suggested that knowledge alone is insufficient to ensure appropriate breast cancer preventive behavior. Instead, behavior is shaped by a combination of accurate knowledge, perceived risk, cultural beliefs, emotional responses, and health system accessibility.

In this study, participants aged 36–40 years were 2.21 times more likely to have good knowledge of breast cancer compared with those aged ≤ 25 years (AOR = 2.21, 95% CI: 1.51–3.14, *p* = 0.002). This finding was consistent with studies done in Ethiopia that reported younger age was linked to poorer knowledge and practices [62] and in Lesotho that reported women aged 45–49 years had significantly higher awareness (AOR = 2.87) [63]. The observed association may be attributed to older women's greater engagement with healthcare services and increased opportunities to receive health education.

In the current study, participants with a family history of breast cancer were 2.13 times more likely to have good knowledge regarding breast cancer compared with those without a family history (AOR = 2.13, 95% CI: 1.24–3.61, *p* = 0.004). Similarly, respondents who had a current breast problem were 2.24 times more likely to demonstrate good knowledge than those without a current breast problem (AOR = 2.24, 95% CI: 1.48–3.84, *p* = 0.004). This finding was similar with studies done in Ethiopia that reported that women with a family history of breast cancer have significantly higher knowledge and are more likely to practice breast self‐examination [64], and absence of family history was linked to poor screening practices (AOR = 5.2 for poor practices) [62]. This may be because personal or familial exposure to breast‐related conditions increases awareness, motivates information seeking, and facilitates interactions with healthcare professionals.

In our study, participants with a family history of breast cancer had higher odds of good practice toward breast cancer detection and prevention compared with those without a family history (AOR = 1.64, 95% CI: 0.96–2.31, *p* = 0.041) and were more likely to have a good attitude toward breast cancer than those without a family history (AOR = 2.35, 95% CI: 1.26–4.46, *p* = 0.004). This finding was similar with studies done in Ethiopia that found participants with a family history had significantly higher preventive practices, knowledge, and positive attitudes toward breast cancer [65], and a history of any breast problem was associated with higher BSE practice (AOR = 1.4, though lower than your 3.54) [66].

In this study, participants with a family history of breast cancer were more likely to have a good attitude toward breast cancer than those without a family history (AOR = 2.35, 95% CI: 1.26–4.46, *p* = 0.004). Likewise, respondents with a current breast problem had significantly higher odds of having good attitude toward breast cancer (AOR = 3.64, 95% CI: 2.62–8.24, *p* = 0.001). participants with a current breast problem were 3.54 times more likely to have a good preventive and detection practices than their counterparts (AOR = 3.54, 95% CI: 1.87–6.25, *p* = 0.003). This finding was similar with studies done in Ethiopia that found women with a family history or breast symptoms show significantly better attitudes toward early detection [67], and current breast problems were among the strongest predictors of both a positive attitude and good practice [68]. These findings emphasized the important role of personal and family experiences with breast‐related conditions in shaping knowledge, attitudes, and preventive practices related to breast cancer. The findings of this study have important implications for public health practice, policy, health education, and future research. The study demonstrated the necessity of strengthening breast cancer awareness programs within workplaces, healthcare facilities, and community settings. Health education initiatives should focus particularly on correcting misconceptions regarding breast cancer risk factors, improving understanding of screening recommendations, and promoting the importance of routine preventive practices regardless of the presence of symptoms. Since mass media was identified as the primary source of information, television, radio, and other media platforms can serve as effective channels for disseminating accurate breast cancer information.

### 4.1 Limitations of the Study

This study has some limitations. It is cross‐sectional, capturing knowledge and practices at a single point. Data were self‐reported, introducing potential recall and social desirability biases, and may not fully reflect actual behaviors. The sample may not represent all Ethiopian women, limiting generalizability. Despite these limitations, the findings provide valuable insights into gaps in breast cancer awareness and practices.

## 5 Conclusion

This study revealed substantial gaps in breast cancer awareness among Ethiopian women. Although common symptoms such as breast pain, swelling, lumps, and breast size changes were widely recognized, knowledge of risk factors, screening recommendations, and proper self‐breast examination procedures remained limited. Awareness of lifestyle‐related risk factors such as smoking, alcohol consumption, and physical inactivity was relatively high, whereas understanding of dietary, reproductive, hormonal, and radiation‐related risks was poor, with notable misconceptions regarding sunlight exposure. Mass media, health workers, and teachers were the primary sources of information. Barriers to screening included fear, absence of symptoms, privacy concerns, and doubts about effectiveness. Women aged 36–40 years, those with a family history of breast cancer, and those with current breast problems were significantly more likely to demonstrate good knowledge, positive attitudes, and preventive practices. Current breast problems emerged as the strongest predictor of favorable attitudes and practices. These findings underscore the need for targeted, culturally appropriate health education and awareness programs that promote early detection, address misconceptions, and emphasize modifiable risk factors through healthcare services, mass media, and educational institutions.

## Author Contributions

**Abaineh Munshea:** conceptualization, investigation, methodology, writing – original draft, formal analysis, writing – review and editing, project administration. **Bizuayehu Kerisew:** conceptualization, investigation, writing – original draft, formal analysis. **Endalkachew Nibret:** conceptualization, investigation, writing – original draft, methodology, writing – review and editing, formal analysis, supervision. **Mastewal Alehegn:** investigation, methodology, formal analysis. **Belaynesh Tazebew:** investigation, methodology, formal analysis. **Alemayehu Abate:** conceptualization, investigation, writing – original draft, methodology, writing – review and editing, formal analysis, validation.

## Funding

The authors have nothing to report.

## Ethics Statement

Ethical approval was obtained from Institutional review board of Bahir Dar University. Written informed consent was ensured from all study participants to take part in the study voluntarily after they get informed about the objective and purpose of the study. This study was performed in accordance with the Declaration of Helsinki. All authors have read and approved the final version of the manuscript Alemayehu Abate had full access to all of the data in this study and takes complete responsibility for the integrity of the data and the accuracy of the data analysis The corresponding author (Alemayehu Abate) affirms that this manuscript is an honest, accurate, and transparent account of the study being reported; that no important aspects of the study have been omitted; and that any discrepancies from the study as planned.

## Conflicts of Interest

The authors declare no conflicts of interest.

## Data Availability

All the generated data in this article are included in the manuscript. The original data can be obtained from the corresponding investigator upon request Alemayehu Abate alexu2love@gmail.com.
